# Percutaneous transluminal angioplasty and stenting vs aggressive medical management on stroke or intracranial atherosclerotic stenosis: a systematic review and meta-analysis

**DOI:** 10.1038/s41598-023-34663-1

**Published:** 2023-05-09

**Authors:** Zhiyu Lai, Mingqiang Peng, Haoming He, Yingbin Li, Xiaoxin Bai, Jun Cai

**Affiliations:** 1 Diagnosis and Treatment Center of Encephalopathy, The Second Affiliated Hospital of Guangzhou University of Chinese Medicine, Guangzhou, 510120 China; 2 Department of Cerebrovascular Surgery, Hospital of Guangzhou University Mega Center, Guangdong Provincial Hospital of Chinese Medicine, Guangzhou, 510006 China; 3 The Second Clinical College of Guangzhou University of Chinese Medicine, Guangzhou, 510120 China; 4 Department of Neurosurgery, Hospital of Guangzhou Higher Education Mega Center, Guangdong Provincial Hospital of Chinese Medicine, No. 55 Neihuan Xi Road, Guangzhou, 510006 Guangdong China

**Keywords:** Diseases, Health care, Medical research, Neurology

## Abstract

There are currently two main treatment strategies mainly for high-risk patients: percutaneous transluminal angioplasty and stenting (PTAS) and aggressive medical management (AMM). However, the choice between PTAS or AMM remains controversial for patients with stroke or intracranial atherosclerotic stenosis (ICAS). The investigators searched the PubMed, Web of Science, Embase, Scopus, and Cochrane library databases. Randomized controlled trial (RCT) comparing PTAS and AMM for patients with stroke or ICAS were selected. RevMan 5.3 was used to analyze the results and assess risk of bias. The primary endpoints are stroke and death within 30 days after enrollment, or ischemic stroke in the territory of the qualifying artery beyond 30 days, and entire follow-up endpoints. The secondary outcomes were the disabling or fatal stroke, and incidence of death within 3 years. Four studies, 989 patients were included in this article. The AMM group was superior in the entire follow-up endpoint (OR 0.56; 95% CI 0.40, 0.79). The AMM also better in primary endpoint within 30 days (OR 0.32; 95% CI 0.17, 0.61). There was no significant difference beyond 30 days (OR 1.08; 95% CI 0.63, 1.86). The remaining outcomes, such as stroke and death, were not significantly different (P > 0.05). This meta-analysis shows AMM is significantly more effective than PTAS in subjects with ICAS due to the high rate of periprocedural stroke (OR 0.32; 95% CI 0.17, 0.61) and stroke during the entire follow-up (OR 0.56; 95% CI 0.40, 0.79) associated with PTAS. Furthermore, PTAS offers no additional benefits over AMM beyond 30 days (OR 1.08; 95% CI 0.63, 1.86).

## Introduction

Stroke is the second-leading cause of death and is the third-leading cause of death and disability combined in worldwide^1^. A systematic analysis noted that Stroke and ischemic heart disease were the leading causes of death in China in 2017^2^.

Intracranial atherosclerosis stenosis (ICAS) is a leading cause of ischemic stroke across the globe^3,4^, and it is known to significantly increase the risk of mortality and disability following stroke and associated with a high risk of recurrent stroke^3,5,6^. In patients with transient ischemic attack (TIA) or stroke, recurrent stroke is at high risk of occurrence even with aspirin and management of vascular risk factors^7,8^. Therefore, the treatment options are crucial to reduce mortality and stroke recurrence.

There is still a challenge in preventing stroke recurrence for patients with conventional medications and risk factor management^9^. This has led to the development of alternative therapies, including percutaneous transluminal angioplasty and stenting (PTAS) and aggressive medical management (AMM). While some studies have shown benefits of PTAS for high-risk patients^10–15^, the choice between PTAS and AMM remains controversial^9^. Several published randomized controlled trials (RCTs) have provided ideas, but there are different conclusions and a lack of high-quality systematic reviews and meta-analysis. This particular systematic review and meta-analysis focused on high-quality RCTs published before October 1, 2022, and aimed to compare the effects of PTAS and AMM on patients with stroke or ICAS.

## Methods

### Study selection

**Figure 1:**
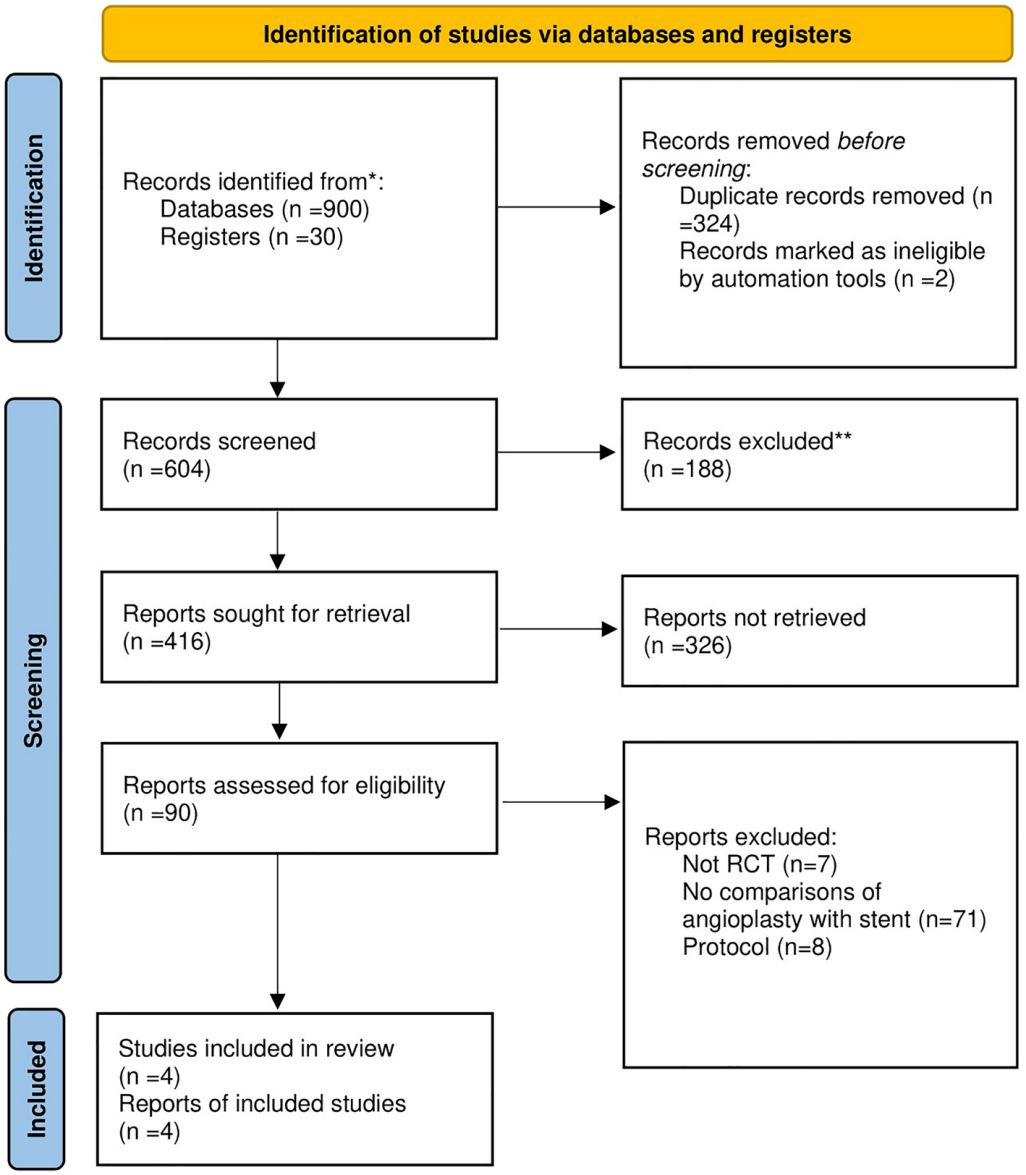
PRISMA flow diagram of search strategy and included studies.

This systematic review and meta-analysis were performed in accordance with the Preferred Reporting Items for Systematic Reviews and Meta-analyses (PRISMA) guidelines^16,17^. The whole retrieval process is shown in Fig. 1. Our research conducted a systematic search on the literature published before October 1, 2022 in PubMed, Web of Science, Scopus, Embase and Cochrane library databases. The search terms are as follow: (balloon angioplasty or PTA or percutaneous transluminal angioplasty or angioplasty) AND (Stenting or stent) AND (cerebral ischemia or strok or cerebral ischemia or intracranial arteriosclerosis or intracranial artery) AND (Randomized controlled trial OR RCT OR randomized OR random OR controlled trials), all restricted to title, abstract, and keywords. After achieving the preliminary retrieval results, researchers screened literature according to the title and abstract independently. Any disagreement was resolved by members that are not involved in study selection. All references were managed via EndNote X9 (Thomson Reuters, NY, USA).

### Eligibility criteria

Studies in this meta-analysis must meet the following criteria: RCT, percutaneous angioplasty and stenting (PTAS) compared with aggressive medical management (AMM), patients undergo cerebral ischemia or stroke. In addition, studies with the following characteristics were excluded: not RCT, patients < 18 years, registered but unpublished research, lack of data required for meta-analysis, no comparisons of angioplasty with stent, and unclear grouping.

### Risk of bias assessment

Based on the cochrane collaboration tool^18^, investigators analyzed the risk of bias of the included studies independently and obtained the overall bias of the studies, which were evaluated with high risk, low risk and unclear. The bias evaluated included: Random sequence generation (selection bias), Allocation concealment (selection bias), Blinding of participants and personnel (performance bias), Blinding of outcome assessment (detection bias), Incomplete outcome data (attrition bias), Selective reporting (reporting bias), Other bias (baseline imbalance, Conflict of interest, etc.). If the results were different, another investigator was required to decide. Assessments were stored and managed in RevMan 5.3(Review Manager. Version 5.3. Copenhagen: The Nordic Cochrane Centre, The Cochrane Collaboration, 2014.). As the blind in this study is difficult, a study with less than 3 high risk was defined as high confidence.

### Data extraction

All data extraction work was done independently. If the results could not be negotiated due to differences in results, the other researcher needs to be asked to reach a consensus. We extracted research data from included studies and stored them into Microsoft Excel Collection Data Sheets. Evaluated Study and Patients’ Characteristics was listed in Table 1.

**Table 1:** Summary of the characteristics of studies and patients in 4 eligible RCTs.

Author yr	Group (n)	Ages, mean ± SD, yr	Gender, M/F	Hypertension, n	History of coronary artery disease, n	History of stroke (not qualifying event), n	Qualifying event, stroke/TIA	Smoker, never/former/current
Gao et al.^9^	PTAS (176)	56.7 ± 9.4	128/48	117	19	ND	89/87	96/39/41
	AMM (182)	55.9 ± 9.8	135/47	125	19	ND	105/77	94/38/50
Miao et al.^20^	PTAS (36)	53.42 ± 13.55	24/12	23	3	ND	7/29	ND/ND/21
	AMM (34)	49.18 ± 9.29	25/9	15	5	ND	8/26	ND/ND/19
Zaidat et al.^19^	PTAS (58)	61.8 ± 12.28	41/17	49	10	ND	36/24	25/22/11
	AMM (53)	61.8 ± 12.82	32/21	43	12	ND	34/22	24/17/12
Derdeyn et al.^15^	PTAS (224)	61.0 ± 10.7	127/97	200	47	60	142/82	90/79/54
	AMM (227)	59.5 ± 11.8	145/82	203	59	58	152/75	78/80/69

*PTAS* percutaneous angioplasty and stenting, *AMM* aggressive medical management, *RCT* randomized control trial, *ND* not declared, *M* male, *F* female, *IQR* interquartile range, *SD* standard deviation, *TIA* transient ischemic attack, *Yr* year.

### Outcomes

The primary outcome of this systematic review consisted of stroke and death within 30 days after enrollment, or ischemic stroke in the territory of the qualifying artery beyond 30 days and entire follow-up endpoints. The secondary outcomes were the stroke in the same territory within 2 and 3 years, disabling or fatal stroke, and incidence of death within 3 years.

### Statistical analysis

RevMan 5.3 was used for all data analysis in this study. We reported the odds ratios (OR) and 95% confidence interval (CI). And we used the Mantel–Haenszel method for analysis. Heterogeneity was assessed before meta-analysis of included studies. When the heterogeneity test P < 0.05 or I^2^ > 50%, the random model is used, otherwise the fixed model is selected.

## Results

### Literature search findings

Our research has been registered with PROSPERO; the registration number is CRD42022362266. We searched in PubMed, Web of Science, Cochrane library, Scopus, and Embase databases on the title, abstract, and keywords of the literature, and 913 articles were obtained (Databases n = 900, Registers n = 30). We used EndNote X9 to find duplicates, and exclude non-clinical studies. Then, we reviewed the abstract and title, 90 clinical studies were included in the final review phase. We screened the full text of these studies, and 4 included studies were finally determined (Not RCT: 7; No comparisons of angioplasty with stent: 71; Study protocol: 8). Literature Search Findings is shown in PRISMA_2020_flow_diagram (Fig. 1).

### Study and patient characteristics

We summarized the study and patient characteristics in Table 1, including author, group, age, gender, medical history (Hypertension, coronary artery disease, and stroke), qualifying event, and smoker. All studies reported the age, sex, hypertension, smoker, and history of coronary artery disease of the patients. Only one study did not reported history of stroke (not qualifying event)^15^.

### Risk of bias assessment and study quality

**Figure 2:**
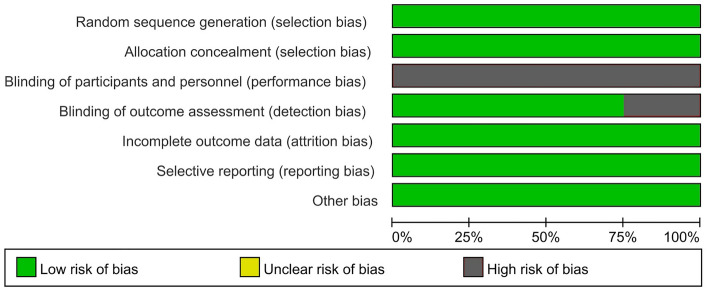
Risk of bias graph: review authors’ judgements about each risk of bias item presented as percentages across all included studies.

**Figure 3:**
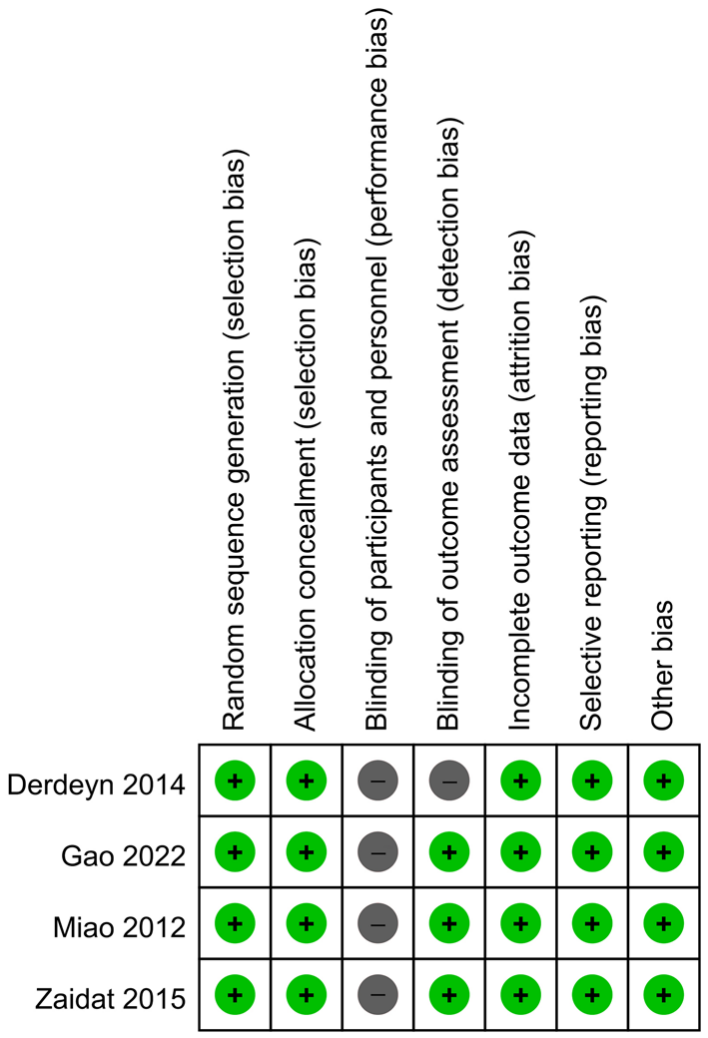
Risk of bias summary: review authors’ judgements about each risk of bias item for each included study.

We used RevMan 5.3 to summarize the bias of the included studies in Figs. 2, 3. It was expressed as high risk, low risk, and unclear. For the included RCTs, design of the blinding of participants and personnel was considered difficult. Only one study defined high risk explicitly reported blinding of outcome assessment^15^. As we only included published RCTs and assessed the risk of bias, all included studies ware defined as high confidence. So, this systematic review and meta-analysis could be seen as “high level” of evidence.

### Primary outcome: entire follow-up endpoint

**Figure 4:**
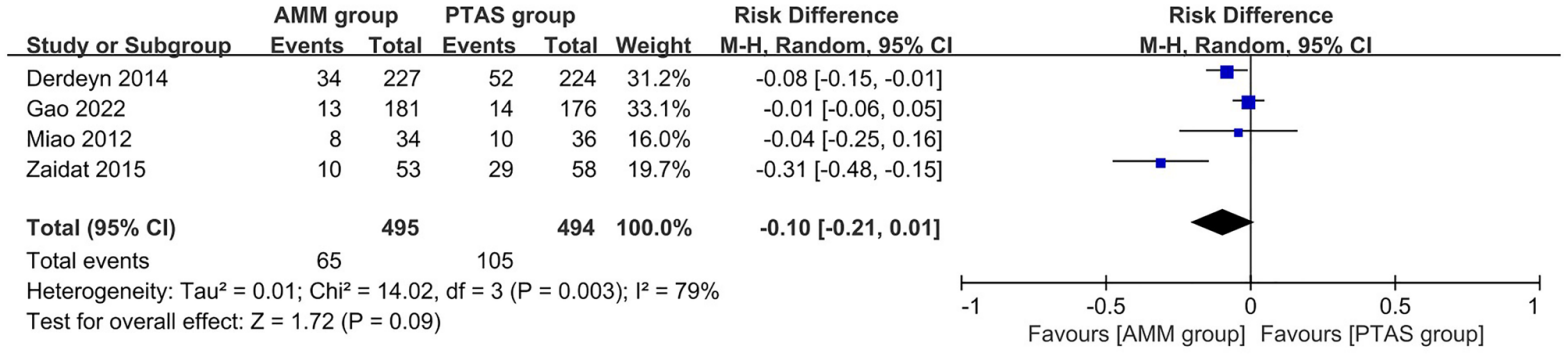
Primary endpoints in entire follow-up.

Four studies^9,15,19,20^ reported the entire follow-up endpoint with a sample size of 989. The pooled OR (95% CI) was 0.56 (0.40, 0.79) in favor of AMM group, heterogeneity test I^2^ = 49% was not significant (Fig. 4). These results reached statistical difference (P = 0.0009).

### Primary outcome: within and beyond 30 days after enrollment events

**Figure 5:**
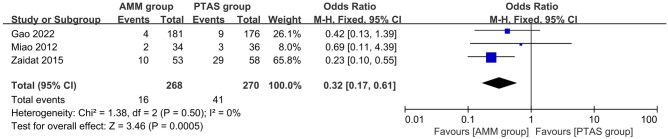
Primary endpoints within 30 days.

Three studies^9,19,20^ reported the primary endpoint within 30 days after enrollment with a sample size of 538. The pooled OR (95% CI) was 0.32 (0.17, 0.61) in favor of AMM group, heterogeneity test I^2^ = 0% was not significant (Fig. 5). These results reached statistical difference (P = 0.0005).

**Figure 6:**
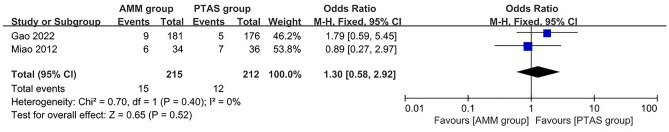
Primary endpoints beyond 30 days.

Two studies^9,20^ reported the primary endpoint beyond 30 days after enrollment with a sample size of 427. The pooled OR (95% CI) was 1.30 (0.58, 2.92), heterogeneity test I^2^ = 0% was not significant (Fig. 6). The difference in results was not significant (P = 0.52).

### Secondary outcomes: stroke events

**Figure 7:**
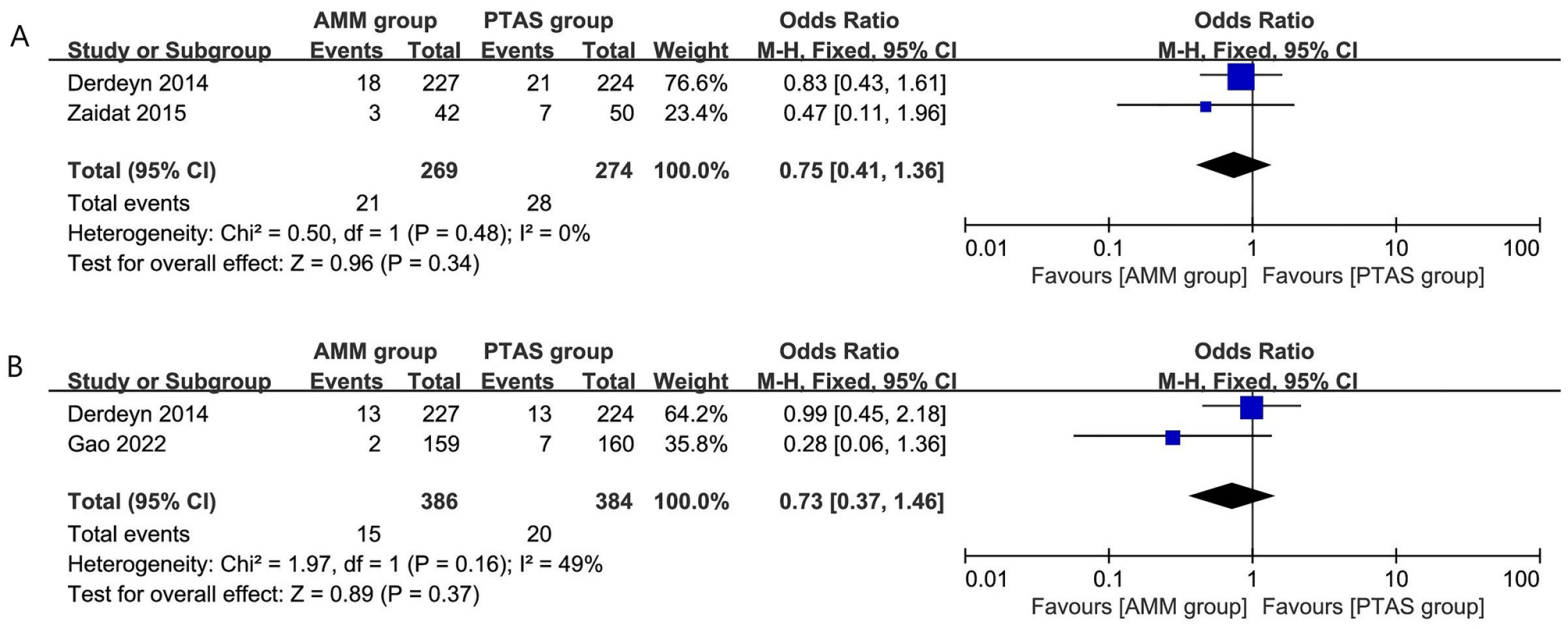
Secondary outcomes: disabling or fatal stroke (**A**) and incidence of death (**B**).

Two studies^15,19^ reported the disabling or fatal stroke with a sample size of 543. The pooled OR (95% CI) was 0.75 (0.41, 1.36), heterogeneity test I^2^ = 0% was not significant (Fig. 7A). The difference in results was not significant (P = 0.34).

### Secondary outcomes: incidence of death

Two studies^9,15^ reported the disabling or fatal stroke with a sample size of 770. The pooled OR (95% CI) was 0.73 (0.37, 1.46), heterogeneity test I^2^ = 49% was not significant (Fig. 7B). The difference in results was not significant (P = 0.37).

## Discussion

This systemic review and meta-analysis in patients presenting with stroke and ICAS demonstrated that primary end points in the AMM group were superior to PTAS group in entire follow-up and within 30 days, and there was no significant difference beyond 30 days. In addition, there was no significant difference in either disabling or fatal stroke or mortality between the two groups.

The study found that AMM was more beneficial than PTAS both during the entire follow-up and within the first 30 days. This is consistent with other studies^9,21^ and suggests that AMM should be the preferred treatment for patients with ICAS^22^. Moreover, it is associated with unstable plaque, which increases the risk of adverse events such as distal embolism after stenting, that AMM is superior to PTAS in entire follow-up and within 30 days^23,24^. Chimowitz et al.^21^ included patients with TIA or nondisabling stroke due to 70–99% stenosis of the diameter of the large intracranial arteries confirmed by angiography within 30 days before enrollment. Similar to the conclusions of this study, AMM provided more benefits for patients than PTAS within 30 days. However, there were no statistically significant differences in any of the outcomes beyond 30 days in our study. The primary endpoint was also assessed in a multicenter, open-label RCT by Gao et al.^9^, which was shown no statistical differences in outcomes beyond 30 days. The variations in findings could result from patient-specific factors, although it's important to note that other biases also play a significant role. Conducting multicenter studies, which involve larger numbers of participants and operators, may help mitigate these factors. The significance of experience is indicated by the correlation between high-volume centers and a reduced risk of complications^25–28^. Furthermore, the results of the PTAS group may be influenced by the effectiveness and limitations of the stent device, and any improvements made to the stent could increase both safety and success rates.

Furthermore, variations in patient selection can also impact outcomes. For instance, Gao et al.^9^ conducted patient screenings using magnetic resonance imaging (MRI) and computed tomography in addition to angiography, therefore excluding those who only had perforator stroke without artery-to-artery embolism or distal hypoperfusion. This technique helps target high-risk patients, enabling better selection for intracranial PTAS and reducing the incidence of perforator occlusion during stent implantation-perforator occlusion has been linked to perioperative embolism in previous studies^29–33^. For the difference between short-term and long-term outcomes, this may be related to the timing of treatment. Early stenting is associated with a higher risk of complications, and longer time intervals have a reduced risk of complications^15,21,27^. In addition, the effect of PTAS may vary depending on the stage of the stroke.

The efficacy of endovascular treatment for ICAS is still controversial, unsatisfactory results and new cerebral ischemic lesions were the main sources of contradictions. The Stenting and Aggressive Medical Management for the Prevention of stroke in Intracranial Stenosis (SAMMPRIS) states that, the 30-day rate of stroke or death in the stenting group was 14.7%^21,29,34^. Hou et al.^35^ conducted a prospective study between April 2020 and July 2021, evaluating thin-section diffusion-weighted MRI and patient characteristics in individuals who underwent endovascular treatment. The study aimed to identify the risk factors associated with the procedure. The findings of the study revealed that there was a high incidence of new cerebral ischemic lesions after endovascular treatment, and smoking and the number of procedures emerged as significant risk factors. It should be noted that the study was based on ICAS participants with maximal drug therapy failure. In addition, stent occlusion within 24 h of endovascular treatment is a common complication after stent implantation. Allard et al.^36^ reported that stent occlusion was observed in 20.9% of patients who underwent stenting for endovascular therapy, and this condition was found to be linked with worse functional outcomes.

The plaque morphology of symptomatic intracranial atherosclerotic lesions is an important factor affecting the therapeutic effect and prognosis. Hou et al.^35^ demonstrated that smoking is a significant predictor of the occurrence of occlusion after endovascular treatment, which may increase the risk of plaque vulnerability^37^. Moreover, it has been suggested that undergoing multiple endovascular treatments could increase the risk of post-treatment occlusion due to a higher susceptibility to plaque rupture and in situ thrombosis. Hypertension is also a risk factor for ICAS. A meta-analysis comprising 17,133 participants established a significant association between hypertension and an elevated risk of ICAS^38^. Li et al.^39^ conducted a retrospective hospital-based multi-center case–control study with the objective of investigating the dose–response relationship between blood pressure and ICAS. The findings of the study revealed that the risk of ICAS rose by 32%, 28%, and 35% for every 10 mmHg increase in SBP, DBP, and PP, respectively. Furthermore, there was a significant increase in the burden of ICAS for every 10 mmHg increase in systolic and pulse pressure. Similarly, several studies have reported similar findings^40,41^. Besides, Wang et al.^42^ demonstrated that stress hyperglycemia is associated with ICAS and increases the risk of recurrent stroke. stress hyperglycemia ratio (SHR) has a better predictive effect than fasting plasma glucose (FPG) and hemoglobin A1c (HbA1c) levels as a biomarker.

A meta-analysis published in 2017 compared the effects of applying PTAS and drug therapy to patients with Symptomatic Intracranial Atherosclerotic Disease^43^. The results showed that medical therapy was superior to PTAS within 30 days, and there was no statistical difference between those beyond 30 days, which was similar to the results of this study. However, the authors included only 3 eligible RCTs with 581 participants, which may affect the stability of the conclusions. Although PTAS has a high short-term complication rate, based on the importance of extending the time window for endovascular embolization and improving reperfusion, PTAS might be considered as a salvage treatment for failed mechanical embolization of large arterial occlusions in the anterior circulation^44–46^.

This is a novel systematic review and meta-analysis comparing AMM with PTAS in ICAS. We conducted a literature quality assessment to select high-quality RCTs, and subsequently performed a meta-analysis of the outcomes of the included studies. We also assessed the impact of heterogeneity on the conclusions. Based on our analysis, the evidence strength of the conclusions was determined to be high. However, this meta-analysis has several limitations to consider. Firstly, the number of studies included is limited as there are fewer RCTs published in English related to our research topic, and we have excluded all retrospective studies. Secondly, heterogeneity is a challenging aspect of meta-analysis to disregard, with factors such as patient characteristics and inclusion and exclusion criteria contributing to heterogeneity. How ever, in this study, the results of the heterogeneity test were acceptable.

## Conclusion

This meta-analysis shows AMM is significantly more effective than PTAS in subjects with ICAS due to the high rate of periprocedural stroke (OR 0.32; 95% CI 0.17, 0.61) and stroke during the entire follow-up (OR 0.56; 95% CI 0.40, 0.79) associated with PTAS. Furthermore, PTAS offers no additional benefits over AMM beyond 30 days (OR 1.08; 95% CI 0.63, 1.86).

## Data Availability

The datasets used and/or analysed during the current study available from the corresponding author on reasonable request.

## Abbreviations

PRISMA: Preferred reporting items for systematic reviews and meta-analyses
OR: Odds ratio
ICAS: Intracranial atherosclerosis stenosis
RCT: Randomized controlled trial
TIA: Transient ischemic attack
PTAS: Percutaneous transluminal angioplasty and stenting
AMM: Aggressive medical management
CI: Confidence interval
MRI: Magnetic resonance imaging
ICAS: Intracranial atherosclerotic stenosis
